# Neurochemical Phenotype of Reelin Immunoreactive Cells in the Piriform Cortex Layer II

**DOI:** 10.3389/fncel.2016.00065

**Published:** 2016-03-10

**Authors:** Hector Carceller, Laura Rovira-Esteban, Juan Nacher, Eero Castrén, Ramon Guirado

**Affiliations:** ^1^Departamento de Biologia Celular, Spanish National Network for Research in Mental Health, CIBERSAM, Fundación Investigación Hospital Clínico de Valencia, INCLIVA, Universitat de Valencia ValenciaValencia, Spain; ^2^Neuroscience Center, University of HelsinkiHelsinki, Finland

**Keywords:** Reelin, Cajal-Retzius cells, PSA-NCAM, piriform cortex, DCX

## Abstract

Reelin, a glycoprotein expressed by Cajal-Retzius neurons throughout the marginal layer of developing neocortex, has been extensively shown to play an important role during brain development, guiding neuronal migration and detachment from radial glia. During the adult life, however, many studies have associated Reelin expression to enhanced neuronal plasticity. Although its mechanism of action in the adult brain remains mostly unknown, Reelin is expressed mainly by a subset of mature interneurons. Here, we confirm the described phenotype of this subpopulation in the adult neocortex. We show that these mature interneurons, although being in close proximity, lack polysialylated neural cell adhesion molecule (PSA-NCAM) expression, a molecule expressed by a subpopulation of mature interneurons, related to brain development and involved in neuronal plasticity of the adult brain as well. However, in the layer II of Piriform cortex there is a high density of cells expressing Reelin whose neurochemical phenotype and connectivity has not been described before. Interestingly, in close proximity to these Reelin expressing cells there is a numerous subpopulation of immature neurons expressing PSA-NCAM and doublecortin (DCX) in this layer of the Piriform cortex. Here, we show that Reelin cells express the neuronal marker Neuronal Nuclei (NeuN), but however the majority of neurons lack markers of mature excitatory or inhibitory neurons. A detail analysis of its morphology indicates these that some of these cells might correspond to semilunar neurons. Interestingly, we found that the majority of these cells express T-box brain 1 (TBR-1) a transcription factor found not only in post-mitotic neurons that differentiate to glutamatergic excitatory neurons but also in Cajal-Retzius cells. We suggest that the function of these Reelin expressing cells might be similar to that of the Cajal-Retzius cells during development, having a role in the maintenance of the immature phenotype of the PSA-NCAM/DCX neurons through its receptors apolipoprotein E receptor 2 (ApoER2) and very low density lipoprotein receptor (VLDLR) in the Piriform cortex layer II during adulthood.

## Introduction

Reelin is a secreted extracellular matrix glycoprotein, identified 20 years ago by the study of the “reeler” mouse (D’Arcangelo et al., 1995). Previous studies of this particular rodent model found that Reelin is expressed and secreted massively during the early stages of the development by the Cajal-Retzius cells on different brain regions like the neocortex, the hippocampus and the cerebellum (Ogawa et al., 1995; Del Río et al., 1997; Soriano and Del Río, 2005). During this phase, Reelin acts as a chemoattractant of the recently generated neurons, allowing for the correct pattern of neuronal migration and lamination of the main structures of the brain. After early development, the expression of Reelin is downregulated, as Cajal-Retzius cells mainly disappear after their developmental function by cell death or are transformed to functional neurons adopting a new phenotype (Schiffmann et al., 1997; Alcántara et al., 1998; Yabut et al., 2007; Frotscher, 2010). Although the study of Reelin has been classically focused on early development, recent studies have showed an emerging role of Reelin in the adult brain, especially in processes related with neural plasticity like modulation of synaptic plasticity, memory and learning processes (Borrell et al., 1999; Weeber et al., 2002; Niu et al., 2008) and disease (Impagnatiello et al., 1998; Hethorn et al., 2015; Lane-Donovan et al., 2015). In the adult rodent brain, Reelin expression is found sparsely across the telencephalon (Alcántara et al., 1998; Pesold et al., 1998; Ramos-Moreno et al., 2006). However, the Piriform cortex displays a very distinct pattern of expression of Reelin in the rodent adult cortex, although the exact phenotype of the Reelin expressing cells in this area remains unknown.

The Piriform cortex receives synaptic input from the olfactory bulb in the layer I; then through its layer III projects widespread along all the telencephalon (Bekkers and Suzuki, 2013). Of special interest is its layer II, where a subpopulation of immature neurons expressing polysialylated neural cell adhesion molecule (PSA-NCAM), doublecortin (DCX) or cyclic nucleotide-gated (CNGA3) can be found (Nacher et al., 2001; Luzzati et al., 2009; Klempin et al., 2011). However, unlike the neurogenic niches of the adult brain (the subventricular zone and the subgranular zone of the dentate gyrus) these cells are mostly generated during early stages of development (Gómez-Climent et al., 2008; Bonfanti and Peretto, 2011; Rubio et al., 2015). Interestingly, the function and fate of these immature neurons in the Piriform cortex layer II remains unknown (Bonfanti and Nacher, 2012).

In order to improve our understanding about the neural population that express Reelin and its relationship with these immature neurons, we have analyzed the neurochemical phenotype of these cells expressing Reelin in the adult neocortex and Piriform cortex.

## Materials and Methods

### Animals and Histological Procedures

Ten male CD-1 mice and three male Sprague-Dawley rats were caged in a standard lighted environment (12 h light/dark cycle) and free access to food and water. All animal experimentation was conducted in accordance with the Directive 2010/63/EU of the European Parliament and of the Council of 22 September 2010 on the protection of animals used for scientific purposes and was approved by the Committee on Bioethics of the Universitat de Valencia and by the Animal Ethical Committee of Southern Finland.

At the age of 4 months old all the animals were deeply anesthetized with sodium pentobarbital (200 mg/Kg). Animals were perfused transcardially; first briefly with saline and then with 4% paraformaldehyde in sodium phosphate buffer 0.1 M, pH 7.4 (PB). The brains were extracted, postfixed for 30 min at 4°C and stored in PB 0.1 M and sodium azide 0.05%. Then, brains were cut in 60 μm thick sections using a vibratome (Leica VT 1000E, Leica). That same procedure was followed with three CD-1 mice pups at 6 days of age after birth (P6).

### Stereotaxic Viral Injections

Six mice were injected with adeno-associated viruses in two different regions of the right hemisphere. First, we injected 1 μL of AAV6-CAG-GFP in the olfactory bulb (Antero-Posterior −4.3 mm and Lateral −1 mm relative to Bregma, Deep −0.8 mm with an angle of −16 degrees) targeting the mitral cell layer. We used this virus as an anterograde tracer, since it allow us to follow the projection of these neurons to the Piriform cortex. Those same animals were also injected in the Piriform cortex of the right hemisphere (AP −0.46 mm and L −2 mm relative to Bregma, and 4.6 mm deep with an angle of −16 degrees) with 1 μL of AAV5-CMV-mCherry targeting its layer II, to study the morphology of those cells expressing Reelin.

### Immunohistochemistry

Free-floating sections were processed as it follows: after washing with phosphate saline buffer (PBS), non-specific bindings were blocked by 10% normal donkey serum (NDS; Abcys), 0.2% Triton-X100 (Sigma) in PBS for 1 h. Sections were then incubated for 48 h at 4°C with different primary antibody cocktails diluted in PBS—0.2% Triton-X100 (see Table 1). After washing, sections were incubated for 2 h at room temperature with different secondary antibody cocktails also diluted in PBS—0.2% Triton-X100 (see Table 1). Finally, sections were washed in PB 0.1 M, mounted on slides and coverslipped using fluorescence mounting medium (Dako).

**Table 1:** **List of primary and secondary antibodies used in the study**.

Anti	Host	Isotype	Dilution	Company
**Primary antibodies**
Reelin	Mouse	Monoclonal IgG1	1:2000	Millipore
TBR1	Rabbit	Polyclonal IgG	1:1000	Abcam
DCX	Rabbit	Polyclonal IgG	1:1000	Abcam
DCX-C18	Goat	Polyclonal IgG	1:250	Santa Cruz
GAD67	Mouse	Monoclonal IgG2a	1:500	Millipore
Glu2R/3	Rabbit	Polyclonal IgG	1:100	Chemicon
PSA-NCAM	Mouse	Monoclonal IgM	1:700	DSHB
CNGA3	Rabbit	Polyclonal IgG	1:2000	Alomone
GFAP	Rabbit	Polyclonal IgG	1:500	Millipore
NeuN	Rabbit	Polyclonal IgG	1:1000	Millipore
VLDL	Rabbit	Polyclonal IgG	1:100	Abcam
APOER2	Rabbit	Polyclonal IgG	1:100	Abcam
**Secondary antibodies**
Goat IgG	Donkey	Alexa555	1:400	Invitrogen
Mouse IgG1	Donkey	DyLight 649	1:400	Jackson
Mouse IgG2A	Donkey	Alexa555	1:400	Invitrogen
Mouse IgM	Donkey	Alexa488	1:400	Invitrogen
Rabbit IgG	Donkey	Alexa555	1:400	Invitrogen
Rabbit IgG	Donkey	Alexa488	1:400	Invitrogen

### Confocal Analysis and Quantification

We analyzed the co-expression of different cell markers in Reelin expressing cells both in the Piriform cortex and the neocortex. We obtained z-stacks of single confocal planes using a confocal microscope (Leica TCS SPE or Zeiss LSM 700). At least three different coronal sections were analyzed per animal between +0.5 and −2.0 mm Antero-Posterior relative to Bregma. From each section 20 cells were analyzed in the Piriform cortex, while in the neocortex all Reelin expressing cells of each stack where analyzed to obtain the percentages describing the neurochemical phenotype, expressed over the total of Reelin expressing cells.

To analyze the animals injected with different adeno-associated viruses, we first obtained single confocal planes at each injection site to confirm the correct location of each injection. Then we obtained z-stacks in the Piriform cortex to study the dendritic arbor of dendrites expressing mCherry and axons expressing green fluorescent protein (GFP).

## Results

### In the Neocortex Reelin is Expressed Mostly by Interneurons

**Figure 1:**
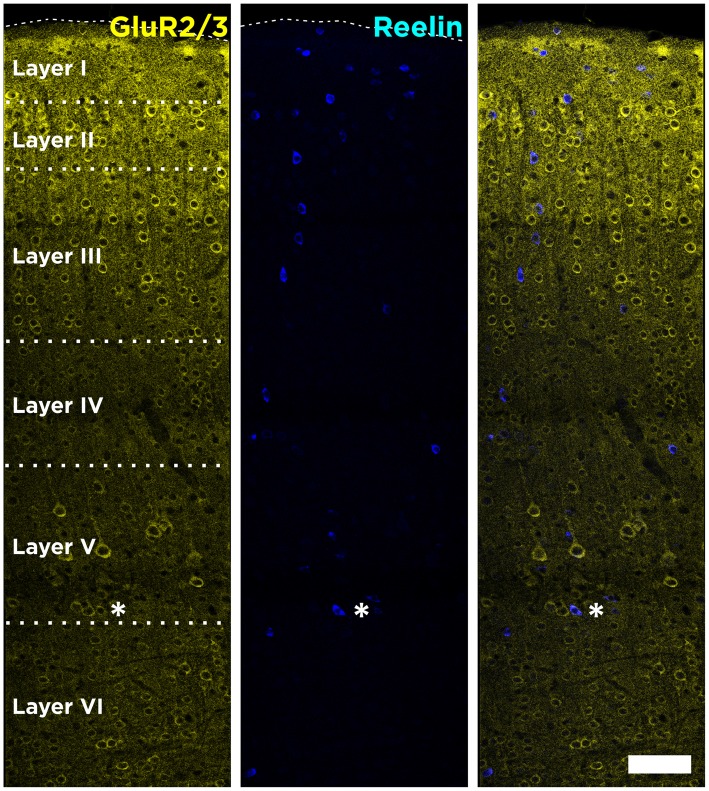
**Distribution of Reelin-positive cells in the neocortex.** Tiled image composition of single confocal planes showing the expression of Reelin and GluR2/3 in all layers of the neocortex of adult mice. GluR2/3 expression defines clearly the boundaries between the different layers. Asterisk indicates a neuron co-expressing Reelin and GluR2/3. Scale bar = 80 μm.

**Figure 2:**
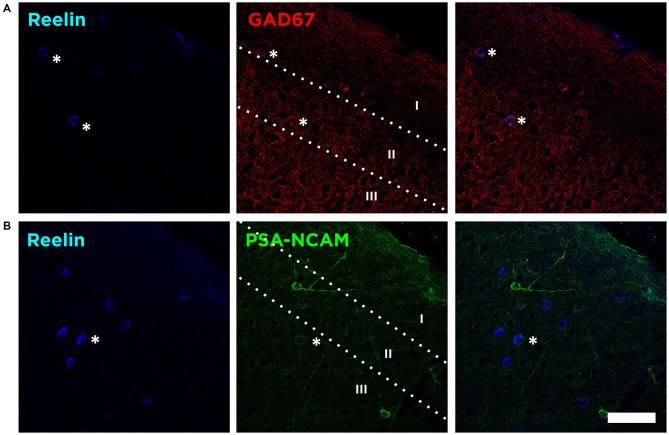
**Reelin is expressed by interneurons lacking PSA-NCAM. (A)** Single confocal planes showing the expression of Reelin, and glutamic acid decarboxylase (GAD67) in the layers I–III of the neocortex. Asterisks indicate co-localization of these markers with Reelin. **(B)** Single confocal planes showing the lack of co-localization of PSA-NCAM and Reelin positive cells in the neocortex. Scale bar = 60 μm.

In the mouse neocortex, Reelin-expressing cells were found scattered in all layers, although with a higher density in the Layer I (Figure 1). When analyzing the neurochemical phenotype of these neocortical Reelin-expressing cells, we have found that few of them (10 ± 2.5%) expressed the Glutamatergic α-amino-3-hydroxy-5-methyl-4-isoxazolepropionic acid (AMPA) receptors 2/3, only expressed in excitatory neurons (Leranth et al., 1996; Toth et al., 1997). On the other hand, most of these Reelin-expressing cells in the neocortex, especially in layer I, expressed the 67 KDa isoform of the glutamic acid decarboxylase enzyme (GAD67; 51 ± 9%), which is exclusively expressed by interneurons (Figure 2A).

We then analyzed whether these Reelin expressing interneurons would also express the PSA-NCAM. We found that the vast majority of Reelin expressing interneurons do not express PSA-NCAM (4.1 ± 2.5%), although in very few cases we found a very weak PSA-NCAM expression in Reelin expressing interneurons (Figure 2B).

### In the Piriform Cortex Layer II Reelin Expressing Cells Lack Mature and Immature Neuronal Markers

**Figure 3:**
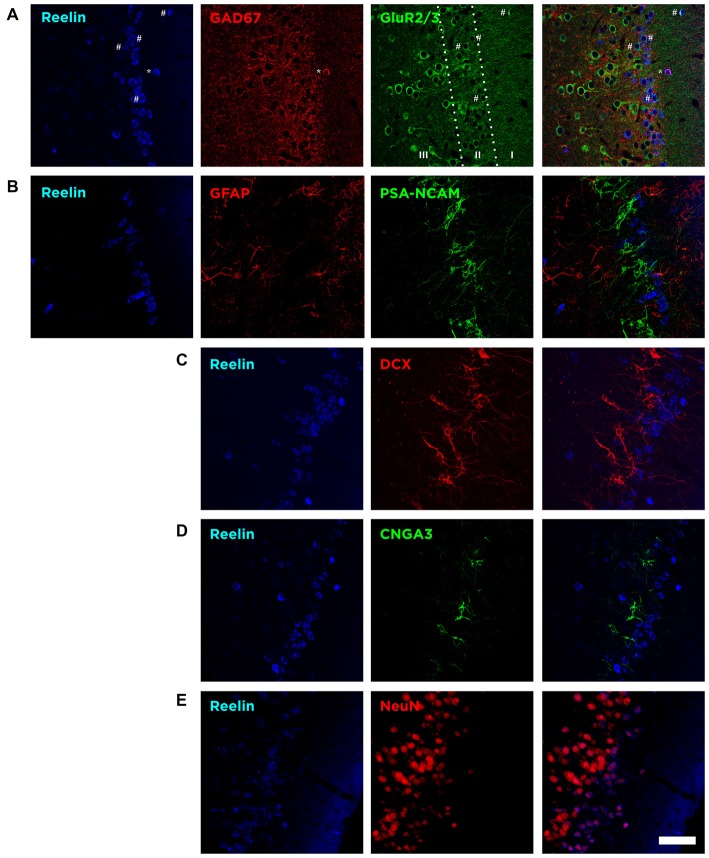
**The majority of Reelin expressing cells in the layer II express Neuronal Nuclei (NeuN) but lack expression of proteins related to mature excitatory and inhibitory neurons, glia and inmmature neurons. (A)** Single confocal planes showing the expression of Reelin, GAD67 and GluR2/3. Reelin expression was particularly high in the uppermost part of the layer II and few Reelin expressing cells expressed either GluR2/3 or GAD67. Hashes indicate Reelin cells expressing GluR2/3 and asterisks indicate the co-expression of Reelin and GAD67. **(B)** Single confocal planes showing Reelin expressing cells lack do not express either PSA-NCAM or glial fibrillary acidic protein (GFAP). **(C)** Single confocal planes showing lack of DCX expression by Reelin expressing cells. **(D)** Single confocal planes showing Reelin expressing cells lack expression of CNGA3. **(E)** Single confocal planes showing the co-localization of NeuN and Reelin positive cells. Scale bar = 60 μm.

In the Piriform cortex, Reelin-expressing cells were densely located in the layer II, being scarce in the layers I and III (Figure 3A). We found that a small fraction of these Reelin expressing cells, mainly located in the layer II, expressed the excitatory marker GluR2/3 (8 ± 3%). On the contrary, in layer I, most of the Reelin expressing cells expressed the interneuronal marker GAD67 (74 ± 14.5%). However, when considering all three layers only few expressed GAD67 (5.8 ± 2.8%; Figure 3A), indicating that most of the cells expressing Reelin in the Piriform cortex layer II expressed neither of these markers.

Then, we analyzed the expression of the glial fibrillary acidic protein (GFAP). We found out that none of these Reelin expressing cells expressed GFAP, therefore we discarded the possibility that these neurons would correspond to glial cells (Figure 3B).

We also analyzed the expression of immature neuronal markers. We found that none of the Reelin expressing cells were immunoreactive for PSA-NCAM, the A3 subunit of the CNGA-3 ion channel (Figures 3B,D) or DCX (Figure 3C).

However, we found that these Reelin expressing cells in the Piriform cortex express the Neuronal Nuclei protein (NeuN) even if in some cells this expression is faint (Figure 3E).

### Reelin Expressing Cells Extend their Dendritic Arbor Towards Layer I and Receive Olfactory Input

**Figure 4:**
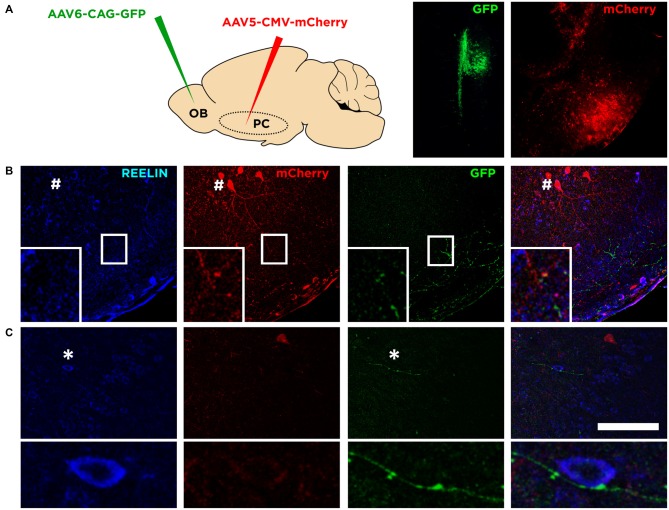
**Connectivity of Reelin expressing cells. (A)** Diagram showing the viral strategy employed and the injection site of each of the virus in the olfactory bulb and the Piriform cortex (left and right pictures respectively). **(B)** Confocal images showing the morphology of a Reelin expressing cell transfected with mCherry (indicated by a hash symbol) and a possible connection between an axonal bouton from the olfactory bulb expressing GFP and a dendrite of a Reelin expressing cell (inset). **(C)** Confocal images showing an axon from the olfactory bulb expressing GFP and a possible contact with a soma expressing Reelin in deep layer II (shown in the inset; indicated by an asterisk symbol). Scale bar = 100 μm.

We analyzed the connectivity of these neurons by transfecting mCherry under a generic promoter (CMV) to the Piriform cortex combined with Reelin immunohistochemistry (Figure 4A). We observed that very few neurons in the upper layer II were transfected. Those expressing mCherry and Reelin displayed a dendritic arbor (Figure 4B) that extended towards the layer I. By transfecting the mitral cell layer of the olfactory bulb with GFP under the CAG promoter (Figure 4A), we could find axons from these cells in the layer I of the Piriform cortex where they appear in apposition to the dendrites of Reelin expressing cells (Figure 4B). All these results suggesting that these neurons expressing mCherry correspond to semilunar neurons. Interestingly however, we found some of these axons from the olfactory bulb innervating deeper layers of the Piriform cortex, contacting perisomatically the Reelin expressing cells located in the layer IIb or III (Figure 4C).

### Reelin Expressing Cells in the Piriform Cortex Layer II Resemble the Cajal-Retzius Cells from the Developing Brain

**Figure 5:**
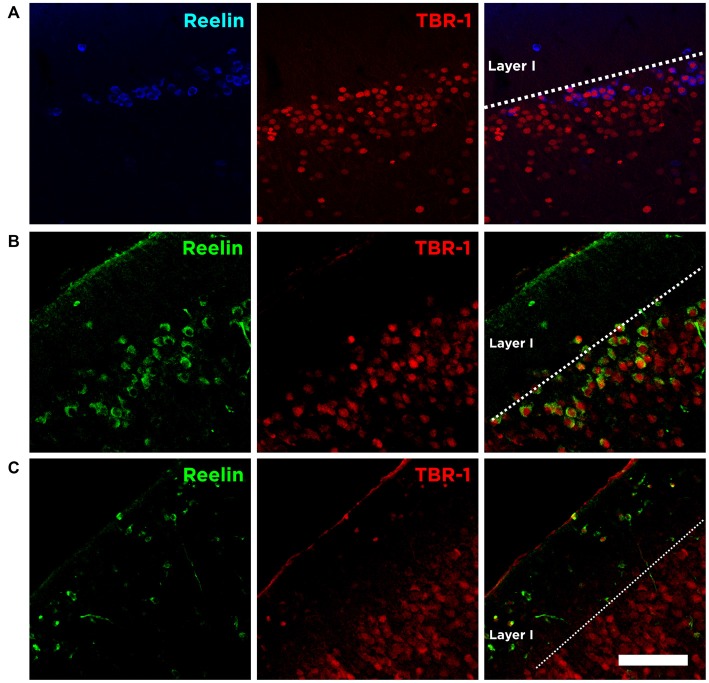
**Expression of Reelin and TBR-1 at different developmental stages. (A)** Single confocal planes showing the expression of Reelin and TBR-1 in the adult Piriform cortex, **(B)** in the developing Piriform cortex (P6) and **(C)** in the developing neocortex. Scale bar = 60 μm.

We analyzed the expression of the neuron-specific transcription factor T-box brain 1 (TBR-1) as it is expressed by neurons of pallial origin and by Cajal-Retzius neurons during brain development (Hevner et al., 2001). Interestingly, we found that the immense majority of Reelin expressing cells in the Piriform cortex layer II express TBR-1 in the adulthood (89.5 ± 2.5%; Figure 5A).

We also analyzed the expression of Reelin and TBR-1 at postnatal brain development (P6). We confirm that Cajal-Retzius cells in the marginal zone of the neocortex express both Reelin and TBR-1 (Figure 5C). Interestingly, we found none or very few Cajal-Retzius cells in the marginal layer of the Piriform cortex at P6. On the other hand, the abundant population of Reelin/TBR-1 neurons in the layer II, which neurochemical phenotype resembles that of Cajal-Retzius cells, is already present at this early stage (Figure 5B).

### Immature Neurons from the Piriform Cortex and the Role of Reelin

**Figure 6:**
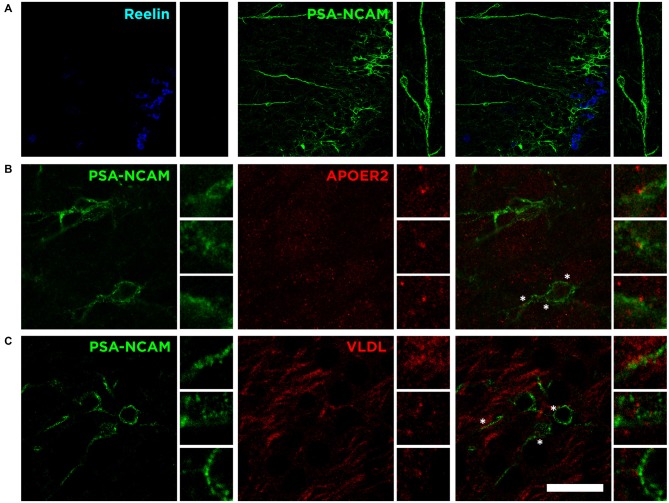
**Possible influence of Reelin on immature elements expressing PSA-NCAM. (A)** Single confocal image showing the expression of Reelin, PSA-NCAM. *Pseudo-radial glia* (inset) expressing PSA-NCAM might act as an scaffolding cortical guide with Reelin secretion at the end, resembling the canonical function of Reelin during development. **(B)** Single confocal plane showing the expression of PSA-NCAM and ApoER2 in the layer II of the Piriform cortex. **(C)** Singles confocal plane showing the expression of PSA-NCAM and VLDL. Asterisks indicate inset where expression of Reelin receptors is in close proximity to PSA-NCAM expressing cells. Scale bar = 60 μm in **(A)** and 20 μm in **(B,C)**.

The detailed analysis of PSA-NCAM expression in the Piriform cortex shows not only the close proximity of both subpopulations: an upper layer II densely packed with Reelin expressing cells and a lower layer II with a numerous immature neurons expressing PSA-NCAM and DCX (Figure 6A). It also reveals the existence of long thick processes forming a *pseudo-radial glia* that expresses PSA-NCAM (Figure 6A), connecting the lowest part of the external capsule and the layer II of the Piriform cortex, in which end we find the Reelin expressing cells (Figure 6A), resembling the canonical function during development of Reelin expressed by Cajal-Retzius cells.

Interestingly, although not limited to the immature neurons of the Piriform cortex, we find expression of both Reelin receptors, apolipoprotein E receptor 2 (ApoER2) and very low density lipoprotein receptor (VLDLR), on the surface of PSA-NCAM expressing neurons in the layer II (Figures 6B,C). These results suggest that the secretion of Reelin might be involved in the control, through these receptors, of the maturation process of the subpopulation expressing PSA-NCAM and DCX in the Piriform cortex layer II.

## Discussion

In the present study, we describe in detail the neurochemical phenotype of Reelin expressing cells in the neocortex and the Piriform cortex in the adult mouse brain. In the neocortex we confirm a widespread distribution of Reelin positive cells among all layers of the neocortex, confirming previous results based in the study of Reelin expression in the adult rodent brain (Alcántara et al., 1998; Pesold et al., 1998; Ramos-Moreno et al., 2006). There are divergent results regarding the nature of these cells according to the species studied. There is solid evidence that Reelin is expressed both by GABAergic and glutamatergic neurons in rats and non-human primates (Pesold et al., 1998; Martínez-Cerdeño and Clascá, 2002; Ramos-Moreno et al., 2006). However, the only previous study that focused in the adult mouse brain found that Reelin is expressed exclusively in GABAergic neurons in the neocortex (Alcántara et al., 1998). We provide here evidence that, at least to a certain extent, there are also few glutamatergic excitatory neurons expressing Reelin in the mouse neocortex.

Reelin is expressed widely during development but is restricted to a subpopulation of neurons in the adult brain (Pesold et al., 1998; Frotscher, 2010). Nevertheless, under different paradigms of adult brain plasticity, there is an increase of Reelin expression suggesting a role in plasticity involving its receptors ApoER2 and VLDL through PSD-95 with N-Methyl-D-aspartate (NMDA) receptors, promoting LTP (Weeber et al., 2002; Hoe et al., 2006; Li et al., 2007; Qiu and Weeber, 2007; Tiraboschi et al., 2013; Hethorn et al., 2015).

Similar to Reelin, PSA-NCAM is expressed by inhibitory neurons in the adult neocortex and it has been suggested to play an important role in adult brain plasticity as well (Nacher et al., 2013). Moreover, both molecules have robustly been related to changes in the synaptic activity of the cells where they are expressed in the adulthood (Qiu and Weeber, 2007; Guirado et al., 2014). Thus, we investigated whether this subpopulation of mature interneurons expressing these molecules might be the same. However, although the distribution of these two subpopulations is remarkably similar, we found that the vast majority of Reelin expressing cells do not express PSA-NCAM.

In the present study, we also analyze the nature of Reelin expressing cells in the Piriform cortex, which has never been studied in detail before. We found Reelin positive cells scattered in the layers I and III and, as previously described, a large density of these cells packed in the layer II (Alcántara et al., 1998). In the layer I, as in the neocortex, Reelin expressing cells also express the inhibitory marker GAD67 indicating that Reelin-positive cells in the Piriform cortex layer I are mainly interneurons, as indicated in that same study (Alcántara et al., 1998), suggesting a common origin with those of the neocortex.

Since the neurons in the layer II lack expression of GAD67 and the large number of cells present there, it was previously assumed these neurons would correspond to excitatory neurons. However, the neurochemical phenotype of these neurons has not ever been investigated. We found that Reelin expressing neurons of the layer II displayed indeed a divergent neurochemical phenotype: most of the Reelin expressing neurons of the layer II expressed neither GAD67 nor GluR2/3. Only few of these neurons express GluR2/3; a marker of excitatory neurons (Leranth et al., 1996). In fact, our viral strategy to study the morphology revealed that the virus did have low preference for the neurons located in the uppermost part of layer II. Only few neurons expressed mCherry and these neurons resembled glutamatergic semilunar neurons, extending dendrites towards the layer I to receive synaptic input from the olfactory bulb, as these semilunar neurons had been previously described (Suzuki and Bekkers, 2011). It is likely then that these semilunar neurons represent the fraction (8%) of Reelin expressing cells expressing GluR2/3 in the layer II of the Piriform cortex.

To explore further the phenotype of these Reelin expressing cells we discarded the possibility that these neurons would correspond to glia, and in fact our results showed that there was no colocalization with GFAP. Then we considered whether Reelin expressing cells might correspond to the immature neurons described in the layer II of paleocortex (Nacher et al., 2002; Luzzati et al., 2009; Klempin et al., 2011). However, our results indicate that these neurons do not express classical immature markers such as PSA-NCAM, CNGA-3 or DCX.

The presence of cortical guide scaffolds expressing PSA-NCAM resembling radial glia intuitively suggests a pathway from subventricular areas to the Piriform cortex as a diversion from the elbow of the rostral migratory stream as suggested previously (Shapiro et al., 2007). However, in a previous study, we demonstrated that these immature neurons expressing PSA-NCAM, CNGA-3 and DCX are generated during embrionary development, and not during adult life (Gómez-Climent et al., 2008). Interestingly, we found that these immature neurons seem to express Reelin receptors such as ApoER2 and VLDL, so we hypothesize that Reelin might play a role in the maturation of these immature neurons. However, there is still controversy whether these neurons mature throughout life: some laboratories have suggested that these neurons mature into interneurons (Xiong et al., 2008; Cai et al., 2009; Zhang et al., 2009) while others have suggested that these neurons mature into excitatory neurons (Gómez-Climent et al., 2008; Luzzati et al., 2009).

Considering these results, we decided to study the expression of the neuron-specific transcription factor TBR-1, a molecule found on pallium-derived neurons committed to differentiate into excitatory neurons and in Cajal-Retzius cells (Hevner et al., 2001; Bedogni et al., 2010). To our surprise we found that the majority of Reelin expressing neurons in the layer II of the Piriform cortex also express TBR-1. Therefore, although the morphology in terms of soma size is different, these Reelin/TBR-1 cells resemble the neurochemical phenotype of Cajal-Retzius cells during development (Hevner et al., 2003). In fact, at postnatal day 6 we observe Cajal-Retzius cells Reelin/TBR-1+ in the marginal layer of the neocortex, but practically absent in the Piriform cortex, where we can find already the Reelin expressing cells in the uppermost part of layer II, suggesting that at that developmental stage the function of the Cajal-Retzius cells is carried out by our subpopulation of Reelin/TBR-1 cells.

Altogether our results indicate that Reelin is expressed in the Piriform cortex layer II by different subpopulations of neurons including, but not limited to, semilunar glutamatergic neurons and interneurons. These results also suggest that Reelin might be involved through ApoER2 and VLDL in the maturation stage of the subpopulation of immature neurons located in the Piriform cortex layer II. Future experiments controlling the secretion of Reelin will be needed to understand yet the physiological role of Reelin in the adult brain.

## Author Contributions

RG designed the study; RG, HC and LR-E performed the experiments; RG and HC analyzed the data; RG, EC and JN wrote the manuscript.

## Funding

This study was supported by ERC Grant No 322742—iPLASTICITY, Sigrid Juselius foundation and Academy of Finland Grant #257486. The Spanish ministry of economy and competitiveness: BFU2012-32512 and MICINN-PIM2010ERN-00577/NEUCONNECT and Generalitat Valenciana: PROMETEO2013/069.

## Conflict of Interest Statement

The authors declare that the research was conducted in the absence of any commercial or financial relationships that could be construed as a potential conflict of interest.
